# Evaluation of Electronic Medical Record (EMR) at Large Urban Primary Care Sexual Health Centre

**DOI:** 10.1371/journal.pone.0060636

**Published:** 2013-04-04

**Authors:** Christopher K. Fairley, Lenka A. Vodstrcil, Sarah Huffam, Rosey Cummings, Marcus Y. Chen, Jun K. Sze, Glenda Fehler, Catriona S. Bradshaw, Tina Schmidt, Karen Berzins, Jane S. Hocking

**Affiliations:** 1 Melbourne Sexual Health Centre, Alfred Health, Melbourne, Victoria, Australia; 2 Sexual Health Unit, School of Population Health, University of Melbourne, Melbourne, Victoria, Australia; 3 Centre for Women's Health, Gender and Society, Melbourne School of Population Health, University of Melbourne, Melbourne, Victoria, Australia; Dana-Farber Cancer Institute, United States of America

## Abstract

**Objective:**

Despite substantial investment in Electronic Medical Record (EMR) systems there has been little research to evaluate them. Our aim was to evaluate changes in efficiency and quality of services after the introduction of a purpose built EMR system, and to assess its acceptability by the doctors, nurses and patients using it.

**Methods:**

We compared a nine month period before and after the introduction of an EMR system in a large sexual health service, audited a sample of records in both periods and undertook anonymous surveys of both staff and patients.

**Results:**

There were 9,752 doctor consultations (in 5,512 consulting hours) in the Paper Medical Record (PMR) period and 9,145 doctor consultations (in 5,176 consulting hours in the EMR period eligible for inclusion in the analysis. There were 5% more consultations per hour seen by doctors in the EMR period compared to the PMR period (rate ratio = 1.05; 95% confidence interval, 1.02, 1.08) after adjusting for type of consultation. The qualitative evaluation of 300 records for each period showed no difference in quality (P>0.17). A survey of clinicians demonstrated that doctors and nurses preferred the EMR system (P<0.01) and a patient survey in each period showed no difference in satisfaction of their care (97% for PMR, 95% for EMR, P = 0.61).

**Conclusion:**

The introduction of an integrated EMR improved efficiency while maintaining the quality of the patient record. The EMR was popular with staff and was not associated with a decline in patient satisfaction in the clinical care provided.

## Introduction

The functions of electronic medical records (EMR) include patient billing, electronic ordering of investigations and receiving investigation results, electronic prescribing, recording of clinical information and in some circumstances, decision support software [1]. They have been widely introduced into medical practices in a number of countries around the world and in the US alone, 27 billion dollars has been allocated to facilitate their introduction [1]. Some countries such as Australia have high rates of EMR adoption with more than 90% of general practices now having some form of EMR and 60% now utilising fully paperless systems [2]. Similar high rates are found in a number of other areas including Scandinavia and New Zealand where all 1,100 general practices use EMR [3]. It is perhaps surprising, considering their widespread use and the enormous financial investment, that relatively few studies have been published evaluating EMR [1].

Synthesizing the published information on EMR is complex for a number of reasons not the least of which is that not all EMR are the same. Some are custom built so they integrate into the entire service, while most are commercial products purchased ‘off the shelf’. These differences are important because custom built EMR systems may be more likely to reduce the time taken for tasks if they are developed around individual practice needs. Another issue that makes interpreting studies of EMR systems difficult is the design of the published evaluations. All are observational studies and hence complicated by systematic differences that exist between clinics or practitioners that choose to use or not use EMR systems. Therefore there is substantial uncertainty about whether observed differences in these studies are due to the EMR system or systematic bias. Finally to accurately evaluate EMR, the data systems need to be equivalent in the EMR and control periods which is rarely the case because by their very nature, the systems often change.

Evaluations of EMR have assessed several different factors including provider satisfaction, patient satisfaction, quality of the services provided or changes in the efficiency of services. However, most published studies evaluating EMR assess only one or two of these factors. In a recent systematic review of the effect of introducing EMRs in primary care none of the studies included all of these factors [4]. Some studies show the introduction of EMR increase efficiency, others show no change or reduce efficiency[4]–[6]. Improvements in efficiency of clinical services is particularly important to the control of sexually transmitted infections because their prevalence is strongly related to access to health care[7], [8].

Melbourne Sexual Health Centre is a large sexual health service that introduced a custom built fully electronic medical record in 2011. We evaluated the effect that the EMR had on the efficiency of the service, the quality of the care provided, the views of staff who used the new system, and finally the satisfaction of patients attending the service. The data systems and collection methods that were used were identical in both the paper and electronic periods.

## Methods

This study was made up of four separate components. The first component was an evaluation of the number of patients seen per hour and testing rates for chlamydia and HIV, before and after the introduction of the EMR (efficiency evaluation). The second component was a qualitative study of completeness and readability of a selection of medical records from both the paper medical record (PMR) and EMR periods (qualitative evaluation). The third component was a survey of the doctors' or nurses' opinions of the PMR and EMR that was carried out after the introduction of the EMR (clinician survey) and the fourth was a survey of the views of patients attending the clinic during the PMR and EMR periods (patient survey).

The Melbourne Sexual Health Centre (MSHC) provides a walk-in clinic[9]. Patients attending the centre initially see a triage nurse and those that are triaged into the service are then classified either as complicated cases who need to see a doctor (e.g. symptomatic) or uncomplicated cases who can see a nurse or doctor (e.g. asymptomatic). Appointments are only available for review within 4 weeks of the initial consultation or for men who have sex with men (MSM) who are able to make appointments for first visits without triage. The centre operates for two 3.5 hour a day (9AM to 12.30 PM, and 1.30 PM to 5 PM), Monday to Thursday but is only open 1.30 PM to 5 PM on Friday. Doctors work in these specific periods but clinic time for nurses is variable and often involves only part of the clinical period as they frequently change roles during the clinic, depending on the workload.

The centre has used software to facilitate patient care since 2002 when it first developed a system written specially for the clinic in Delphi programming language that is called the Clinic Patient Management System (CPMS). In 2002 this software recorded patient demographic details, sexual risk data and also monitored the movement of patients around the clinic, through triage, consultations and investigations and treatments using a spread sheet format on all computer screens. A paper based record was used for all other functions. In 2006 the software (CPMS) was updated to include electronic ordering and receiving results of swab investigations (e.g. gonorrhoea/chlamydia), and electronic prescribing of medications. The results of serological investigations could be viewed electronically but had to be ordered using a hand written paper request form. In June 2008 MSHC implemented computer assisted self interviewing (CASI) using computer touch screens that were operational over the entire evaluation period for this study. All other functions were carried out using a paper record (table 1). From June 2008 until March 2011 the output from CASI was printed onto a paper sticker that was placed in the paper medical record before the patient was seen by a clinician.

**Table 1:** Description of the paper record and electronic record functions.

Function	Paper Record period	Electronic Record period
Client registration	Touch screen by patient	Touch screen by patient
CASI	Touch screen by patient	Touch screen by patient
Patient record	Paper record	Electronic record
Clinical history	Hand written notes	Typed notes
Ordering swab investigations	Click on options in the electronic file, sent electronically to laboratory	Click on options in the electronic file, sent electronically to laboratory
Ordering blood investigations	Hand written paper slip manually taken to blood room	Click on options in the electronic file, sent electronically to blood room (no printing)
Pap smear requests	Hand written slip	Prefilled electronic form that is printed
Injectable medication orders #	Hand written order that is manually taken to blood room	Click on options in the electronic file, sent electronically to blood room (no printing)
Results of blood and swab investigations	Electronic	Electronic
Prescription	Electronic	Electronic

# refers to injectable drugs (e.g. bezathaine penicillin, ceftriaxone).

CASI = computer assisted self interviewing. The electronic medical record period began on the 9^th^ of March 2009.

On the 9^th^ of March 2011 CPMS was further upgraded to function as a fully electronic health record, and for the purposes of this study the EMR period is deemed to begin on this date. The EMR included features that sent blood and injectable medication requests electronically to the procedure room rather than needing to manually take paper requests there. The functions of the EMR are described in table one and compared to the paper record it replaced.

### Efficiency evaluation

#### Number of patients seen per hour

To assess whether the introduction of the EMR was associated with a change in the rate at which patients were seen in the clinic, we compared the number of patients seen by doctors per hour in the 9 months before the introduction of the EMR (1^st^ June 2010 to 8th of March 2011) to the same 9 calendar months after the introduction of the EMR (1^st^ of June 2011 to 8^th^ of March 2012). We did not include the first three months after the EMR was introduced to allow staff an adjustment period. Patients seen by doctors who did not work in both the EMR and PMR periods were excluded from the analysis. Patients seen by nurses were also excluded from this analysis because they do not work fixed clinical times and roles within this period also vary (e.g. may shift to triage nurse role when the clinic is busy, move to the venepuncture or serology room, or only work part of the clinical shift).

We used a general linear model with a Poisson family and a log link function to generate rate ratios to assess differences in the number of patients seen per hour between PMR and EMR. This analysis was adjusted for factors that were likely to influence the duration of a consultation (gender of patient, type of patient (new or returning) and consult type (complicated, uncomplicated, appointment)[10]. Analyses accounted for repeat measures from individual doctors. All analysis was conducted using Stata Version 12.

#### Testing rates

We assessed the proportion of new clients attending the centre who had chlamydia and HIV tests performed. Analyses were conducted separately for males and females and among men, separately for homosexual and heterosexual men. Patients were excluded from the analysis if the doctor or nurse who saw them did not work in both the PMR and EMR period. Odds ratios were calculated using logistic regression to estimate the association between the proportion of patients who had a chlamydia test (any site), an HIV test or an anal swab performed for men with male sexual partners. Robust standard errors were calculated to account for potential intra-cluster correlation by repeated measures from individual doctor or nurse.

### Qualitative Evaluation

For clinicians who worked in both PMR and EMR periods, we examined five patient records from each period. For doctors, only complex patients (symptomatic) were chosen and for nurses, uncomplicated (asymptomatic) patients were chosen. Only records of new patients who attended MHSC for the first time were eligible and only information recorded on the first visit was assessed. We selected the first three new male and two new female patients in each period for each doctor or nurse in the month of November 2010 (PMR) and 2011 (EMR). Files from patients who presented after 4pm were excluded because they could potentially have been briefer because of reduced available consulting time.

Each file was reviewed by one of two clinicians (KB and TS) who were senior doctors with more than 10 years experience at MSHC and who were familiar with both PMR and EMR. Both had conducted quality audits of medical records previously. The first 10% of files were scored by both reviewers and any files allocated different scores were discussed and a consensus reached to further standardize scoring [11].

We used a number of outcome measures for the qualitative evaluation. Firstly the reviewers were asked to measure with a stopwatch the time it took them to develop sufficient understanding of the contents of the initial consultation so they felt they could comfortably proceed to a hypothetical second consultation. Secondly they scored the records of the consultation in relation to a number of different measures; decipherability, quality of the documentation in the history, examination, and management sections of the records, and then provide an overall quality of the documentation. Decipherability was defined as being able to fully understand all that was written. Documentation of duration of symptoms, and recording the presence or absence of drug allergies were also included. The overall quality of the consultation was also assessed. The PMR and EMR were reviewed in alternating order and the assessments of all 10 files from the same clinician were performed in the one sitting.

### Clinician survey

We conducted a staff survey in May 2011, two months after the introduction of the EMR, when we expected that staff had become familiar with the EMR but still had a clear memory of using the PMR. Only staff members that had worked on both the PMR and EMR were eligible for the survey. All eligible MSHC doctors and nurses were provided with a link to the online questionnaire using SurveyMonkey™ (www.surveymonkey.com) together with a letter explaining that their answers were anonymous.

They were asked which file type they preferred for different clinic functions, what effect that the EMR had had on the clinical history and clinical processes and whether they would recommend it to another clinic. We specifically asked doctors and nurses which parts of the consultation and clinical processes they felt were quicker or slower with the EMR.

### Patient survey

For one week in November every year, MSHC undertakes a satisfaction survey which is offered to all clinic attendees. The results are published on the clinic's website (http://www.mshc.org.au/general/MSHCReports/SatisfactionSurveys/tabid/213/Default.aspx). The satisfaction survey was conducted between the 8^th^ and 12^th^ of November in 2010 (PMR period) and between the 7^th^ and 11^th^ of November in 2011 (EMR period). The majority of the survey questions were the same from one year to the next and included questions about the overall satisfaction of the visit and specific questions about the quality of the consultation.

A one sample signed rank test was used to investigate whether the responses obtained on the clinician survey were statistically different from the responses that might have been expected by chance alone.

### Ethics Statement

Ethical approval was granted by the Alfred Hospital Research Ethics Committee (certificate 540/11) for this study. Approval was given not to obtain consent from patients for review of their existing medical records. The ethics committee approved not obtaining written or verbal consent to preserve the anonymity of participants who consent was implied by participation in the survey.

## Results

### Efficiency evaluation

There were 12,560 consultations in the PMR period and 12,669 consultations in the EMR period by 33 different doctors. Twenty two of these doctors worked in both PMR and EMR periods and saw 9,752 consultations in 5,513 hours in the PMR period and 9,145 consultations in 5,162 hours in the EMR period. After adjusting for the type of consultation, sex of the patient and whether they were new or returning patients and the clustering of the doctor, doctors saw 5% more clients during the EMR period compared to the PMR period (adjusted ratio 1.05, 95% confidence interval 1.02–1.08, P = 0.001).

The proportion of consultations for new patients where a chlamydia or HIV test was conducted and proportion of MSM who had an anal swab performed are shown in table 2. Only chlamydia testing in women was significantly different between the two periods. During the PMR period 92% of women received a chlamydia test compared with 88% (P = 0.001) during the EMR period.

**Table 2:** Proportion of new clients tested for chlamydia or HIV by risk group and period.

		*Paper MR*	*Electronic MR*		
*Patient group*	*Test*	n/N (%)	n/N (%)	OR (95% CI)^a^	*p-value* ^a^
Heterosexual men	Chlamydia	1911/2198 (87)	1869/2190 (85)	0.87 (0.74–1.04)	0.11
	HIV	1041/2185 (48)	1033/2174 (48)	1.00 (0.84–1.19)	0.98
MSM	Chlamydia	895/944 (95)	915/972 (94)	0.88 (0.51–1.52)	0.65
	Anal swab	784/944 (83)	820/972 (84)	1.10 (0.84–1.44)	0.49
	HIV	635/918 (69)	677/937 (72)	1.24 (0.98–1.56)	0.07
Female	Chlamydia	1922/2094 (92)	1618/1842 (88)	0.65 (0.50–0.84)	0.001
	HIV	1059/2090 (51)	927/1835 (51)	0.99 (0.83–1.28)	0.94

CI = confidence interval.

MR = medical record period.

N = number of new patients in the patient group. n = number of new patients in the patient group who had the test done.

MSM = men who have sex with men.

### Qualitative evaluation

Thirty doctors or nurses worked in the month of November in both periods and each had 10 files assessed. All measures of quality were high in both the paper and electronic records. A lower proportion of PMR were fully decipherable (89%) compared with EMR (100%, P<0.01), but other measures of quality of management were the same between the record types (P = 0.17). The proportion of the 146 PMR records and 149 EMR graded as good or optimal for the quality of history (94% vs. 95%, P = 0.99), quality of the examination (95% vs. 98%, P = 0.38), quality of management (93% vs. 97%, P = 0.17), and overall quality (97% vs 97%, P = 0.5) was similar respectively. The mean word counts were lower in PMR (65 words) than in EMR (113 words) (P<0.01) but the time for the auditing clinicians to develop sufficient understanding of the patient record was similar (36 vs. 37 seconds, P = 0.34) after adjusting for the complexity of the patient and the clinician who saw them.

### Clinician survey

Of the 44 doctors or nurses who worked during both the PMR and EMR periods, 39 were working during the survey period and were provided with a link to the online survey. Of these 39 eligible doctors or nurses, all responded to the survey (23 doctors and 16 nurses) (Table 3). About half had used an EMR elsewhere, 28% touch typed and half were 45 or older. In comparison to the PMR, more doctors and nurses felt that the EMR provided high quality documentation (P<0.01), and was easier to use (P<0.01). In comparison to the PMR they felt the EMR was faster for selecting patients from the waiting area (P<0.01), for ordering serology and/or injectable medication (P<0.01) but not for completing the medical history (P = 0.43). They also felt that EMR reduced contact between staff during clinical work (P<0.01), and reduced patient eye contact (P<0.01) but despite this thought overall that the EMR did not adversely affect patient rapport (p = 0.47). Overall, 68% preferred the EMR, 89% were satisfied with the EMR and 83% would recommend EMR to other clinics. There were no significant differences in the views of doctors or nurses in the above questions.

**Table 3:** Clinician survey.

Questions	n	%	P value*
***Has the electronic record changed the way you perform your consultation***			
Yes	21	57	0.42
No	16	43	
			
***Has electronic record changed eye contact with patients***			
More eye contact electronic	3	8	<0.01
Same eye contact	21	54	
Less eye contact electronic	15	38	
			
***How has electronic record impacted on interaction with other staff***			
Interact more	3	8	<0.01
interact same	14	38	
interact less	20	54	
			
***Which file type do you prefer for conducting consults***			
Strongly prefer or prefer electronic	25	68	<0.01
Same	6	16	
Strongly prefer or prefer paper	6	16	
			
***Which file type do you prefer for obtaining medical history***			
Strongly prefer or prefer electronic	25	68	<0.01
Same	8	22	
Strongly prefer or prefer paper	4	11	
		
***Which file type do you prefer for best quality consult***			
Strongly prefer electronic	22	60	<0.01
Same	12	32	
Prefer paper	3	8	
			
***Which file type do you prefer for highest quality documentation***			
Strongly prefer or prefer electronic	31	83	<0.01
Same	2	5	
Strongly prefer or prefer paper	4	11	
			
***Which file type do you prefer for building rapport with clients***			
Strongly prefer or prefer electronic	7	19	0.47
Same	20	54	
Strongly prefer or prefer paper	10	27	
			
***Which file type do you prefer for ease of use***			
Strongly prefer or prefer electronic	25	68	<0.01
Same	8	22	
Strongly prefer or prefer paper	4	11	
			
***Which file type do you prefer for communicating with serology***			
Strongly prefer or prefer electronic	27	73	<0.01
Same	5	14	
Strongly prefer or prefer paper	5	13	
			
***Which is faster for selecting client from waiting room***			
Electronic much faster or faster	24	67	<0.01
Same	7	19	
Paper much faster or faster	5	14	
			
***Which is faster for completing clinical history***			
Electronic much faster or faster	15	42	0.43
Same	10	28	
Paper much faster or faster	11	31	
			
***Which is faster for ordering serology***			
Electronic much faster	28	78	<0.01
Same	6	17	
Paper faster	2	6	
			
***Which is faster for ordering injectables***			
Electronic much faster	22	63	<0.01
Same	8	23	
Paper faster	5	14	
			
***Would you recommend electronic to another clinic***			
Strongly recommend or recommend	29	83	<0.01
neither recommend nor not recommend	6	17	
Strongly not recommend or not recommend	0	0	
			
***Satisfaction with electronic***			
Very satisfied or satisfied	32	89	<0.01
Neutral	2	6	
Very unsatisfied or unsatisfied	2	6	

One sample signed rank test.

39 clinicians were sent the survey. There the numbers add up to less than 39, the question was not answered by all clinicians.

### Patient survey

During the PMR period the survey was given to 624 patients of whom 273 (44%) completed it. During the EMR period the survey was given to 790 patients of whom 200 (31%) completed it.

Table 4 shows the results of the satisfaction surveys that were carried out in the PMR period and EMR periods. During both periods the patient satisfaction was high and there were no statistically significant differences in the satisfaction of patients between these two periods (P>0.21). Patients attending during the EMR were asked if they thought that the EMR had affected the quality of their consultation on the day of their visit and only 3% felt that it was worse because of the EMR.

**Table 4:** Patient responses to the annual satisfaction survey in the Paper period (2010) and Electronic period (2011).

Question	Paper period N (%)	Electronic period N (%)	OR (95% CI)
The practitioner made me feel comfortable to discuss sexual health matters			
Strongly agree or agree	255 (99)	178 (97%)	0.28 (0.04, 1.57)
Not sure, disagree or strong disagree	2	5	(P = 0.21)
I did have the opportunity to ask questions- % agree			
Strongly agree or agree	250 (97)	177 (95)	0.63 (0.22, 1.82)
Not sure, disagree or strong disagree	8	9	(P = 0.49)
Thinking about the consultation you had today with our new electronic health record. How do you think the electronic record affected the quality of your consultation today?			
Improved	NA	116 (66%)	(59–73%)
No difference		56 (32%)	(25–39%)
Worse		5 (3%)	(1–6%)
Overall, I am satisfied with the services at MSHC			
Very satisfied, satisfied	231 (97)	176 (96)	0.67 (0.21, 2.1)
Unsure, dissatisfied, very dissatisfied	7	8	(P = 0.61)
If the need arose, I would attend MSHC again			
Yes	229 (97)	180 (98)	1.83 (0.42, 9.1)
No or not sure	7	3	(P = 0.58)

OR = odds ration, CI = confidence interval.

## Discussion

In this study we showed that the introduction of an integrated EMR was popular with staff, was associated with a 5% increase in clinic efficiency among patients seen by doctors and importantly did not change the quality of the medical record or the satisfaction of patients with the service. The majority of practitioners felt that the EMR was easier to use and they also identified specific clinic processes they felt were faster with the EMR, which was consistent with our findings. Unlike some other studies in this area, our study design controlled for doctors' individual characteristics by only including doctors who worked in both periods, thus limiting potential bias. The survey data suggested that the efficiency resulted from reduced time for clinical processes other than completion of the clinical information. If these clinical processes were the cause of the increased efficiency, then encouraging careful analysis of the processes that take time with clinical services is important when designing EMR, particularly for large clinical services that have the capacity to at least in part, design their own systems, or modify ‘off the shelf’ products.

There are a number of factors that need to be considered when interpreting our results. Firstly, this was an observational study and was therefore subject to a number of potential biases. For example there may have been changes in the clinic over this time that we did not measure that could have influenced the results, although every effort was made to identify factors associated with consultation time. In our analysis we negated the practitioner effect by only including practitioners who worked in both periods, but it is possible that those doctors or nurses who were excluded from the analysis may have responded differently if they had worked in both time periods. We also adjusted for the complexity of the patient, and the sex of the patient which can influence the duration of a consultation, and finally we included the same periods of the year, to negate seasonal changes in clinic demand. While we acknowledge there may be other unmeasured factors, all efforts were taken to minimise this.

It is interesting to consider what aspects of the EMR might have contributed to the reduction in time taken to see a patient. The practitioners felt that a number of clinic processes saved them time including a quicker process for collecting patients from the waiting area, electronically ordering and electronically sending requests for serological tests to the venepuncture/procedure room. Instead of labelling and hand writing a request slip or an order for injectable medication, and then walking to the procedure room to leave the request, these processes occurred electronically without the need to print any forms or leave the consulting room. They also reported less interaction with clinic staff which may have also saved time. As the average savings for doctors was only in the order of about 1–2 minutes per consultation it is plausible that these changes were the cause of the greater number of consultations seen per hour. Several other observations suggest that the increased efficiency was predominantly related to increased efficiency of clinic processes including the staff survey that indicated that EMR was not thought to be faster for completing the clinical history.

There is an important implication from the perceptions of doctors and nurses that it was the clinic processes that were responsible for the improved efficiency, rather than completing the clinical details in the EMR alone. Namely, if efficiency is to be driven from an EMR then it needs to be integrated into clinic functions and this may be more difficult to achieve with small clinics that may choose to purchase a commercial product for the storage of clinical notes. This may also explain the diversity of findings in other studies which have provided conflicting results in relation to efficiency[4].

It is also possible that our study underestimated the potential time savings associated with an EMR because a number of functions common to some EMR were already operating at our clinic before the final version was introduced in March 2011 (Table 1). Specifically electronic prescribing, electronic ordering of swab investigations and the electronic return of investigation results were already in place when the final version was introduced. Therefore the 5% time saving that we observed could have been greater if all these features were changed at the same time.

A number of studies have specifically looked at clinical efficiency associated with an EMR. Furukawa undertook a cross sectional analysis of office based physicians in the US and found that those who use an EMR provided 11% more patient consultations per unit time than those who did not (P<0.001)[12]. The authors of this study made the point that it was not a longitudinal analysis and they may not have adjusted for all the differences between patients and practitioners. Specifically it compared practitioners who had chosen to use EMR versus those who had not.

Cheriff, compared changes over time between clinicians adopting a commercial EMR over a six year period between 2001 and 2006 and found that there was an 8% increase in patients seen by clinicians who moved to an EMR compared to those who did not[13]. The authors make the point that there may be systematic differences between those who do and do not adopt to use EMR and that because of the relatively long time period of the study, there may have been other changes occurring to explain the findings[13].

Patil and others undertook a before and after study in a group Urology practice and found no difference in patient encounters before and after the introduction of an EMR[14]. Finally a recent systematic review by Holroyd-Leduc, reported data on a number of studies that did also not show differences between the efficiency of EMR and PMR[4].

There have been a number of studies looking at the differences in the structure and contents of a consultation preformed using an EMR compared to PMR. In general these studies show that EMR have significantly more words than PMR but despite more words, there were not differences in specific elements of the record (e.g. documenting the reason for the consultation) [4], [6]. These findings are consistent with our observation that while the word count almost doubled, there was no evidence of a change in quality.

We did make the observation that there was a statistically significant fall in females tested for chlamydia from 92% of new patients in the PMR period to 88% during the EMR period. No other testing significantly changed. We are unable to explain this small, and probably clinically unimportant, change in testing. The process for ordering chlamydia was the same between the two periods (Table 1) and CASI, which is the tool used to identify risk was also present during both periods hence we think it is unlikely that the EMR was responsible for this change, however we cannot rule out this explanation.

Only three studies were identified in the systematic review published in 2011 included patient views about the impact of introducing an EMR[15]–[17]. All three studies found no adverse effect on the overall quality of consultations associated with the introduction of an EMR, but some studies noted minor differences in interactions, such as reduced time looking at the patients[16]. This is consistent with our findings that the patient's perceptions of the quality of their consultation at MSHC remained high and was not significantly different between paper and electronic records.

There have been a number of different studies that have assessed the views of clinicians about EMR in the US, Norway, Australia and the UK[18]–[21]. These studies found that while there was some initial concern about the introduction of an EMR, this waned with time and most clinicians felt that EMRs improved quality of consultations and saved time[18]–[21]. Negative findings were mainly related to features of the design of the EMR or technical issues relating to IT system failures[18]–[21]. These findings are similar to our study although our EMR was designed with substantial input from clinicians and so a more favourable view may have been expected than from an off the shelf product.
